# Genome Sequence of VanLee, a Singleton Actinobacteriophage That Infects Multiple *Gordonia* Strains

**DOI:** 10.1128/MRA.00519-21

**Published:** 2021-07-29

**Authors:** Vanessa Franco, Kaylee Barnhill, Abbigail Biggs, Jackson Bland, Harris Choudhary, Trey Crogan, Alyssa Finocchiaro, Thomas Fuller, Christopher Hanwacker, Zoe Howard, Mohammed Iqbal, Ann Mathew, Sydney Miller, Shivani Padhye, Emily Rainey, Arianna Rodriguez, Emma Stewart, Michael Chase, Louis Otero, Richard S. Pollenz

**Affiliations:** a Department of Cell Biology, Microbiology, and Molecular Biology, University of South Florida, Tampa, Florida, USA; DOE Joint Genome Institute

## Abstract

VanLee is a singleton phage that was isolated from soil in Florida using Gordonia rubripertincta NRRL B-16540 as the host. The genome is 84,560 bp and has a GC content of 67.8%. VanLee has 164 predicted protein-coding genes and one tRNA. VanLee can infect Gordonia terrae with the same efficiency as G. rubripertincta.

## ANNOUNCEMENT

Bacteriophages provide a rich reservoir of uncharacterized genes and have been critical for studying the evolution and adaptation of phage and bacterial defense systems (1). To isolate evolutionarily diverse actinobacteriophages, the Science Education Alliance-Phage Hunters Advancing Genomics and Evolutionary Science (SEA-PHAGES) program (2) utilizes eight different host actinobacteria (3).

VanLee was isolated from a moist soil sample from Tampa, Florida (28.063333N, 82.411389W), using Gordonia rubripertincta NRRL B-16540 as the host. All bacterial hosts used in this study were grown at 30°C utilizing peptone-yeast-calcium agar (PYCa). Genomic DNA was isolated from a high-titer phage lysate after three rounds of plaque purification using the Wizard DNA cleanup kit (A7280; Promega). Genomic DNA was used to create sequencing libraries with the NEBNext Ultra II FS DNA library preparation kit. Sequencing was performed by the Pittsburgh Bacteriophage Institute, and the library was run on an Illumina MiSeq instrument, yielding 889,244 paired-end 150-bp reads with 1,488-fold coverage. Raw reads were assembled with Newbler (v2.9) (4), yielding a single phage contig. The results were checked for completeness, accuracy, and genome termini using Consed (5). Default parameters were used for all software unless otherwise specified. VanLee is circularly permuted and was bioinformatically linearized such that base 1 is assigned in accord with other *Gordonia* phages (6). VanLee was autoannotated using DNA Master (v5.23.6) (7), and all of the genes were then manually validated for correct starts and predicted functions for the protein products. GeneMark (v2.5) (8) and Glimmer (v3.02) (9) were utilized to assess start sites and coding potential, and Starterator (v1.2) (3) was used to summarize the starts across each family of phage genes. To collect evidence for the gene function and the validity of each gene product, HHpred (v3.2) (10), NCBI BLASTp (11), the Conserved Domain Database (12), TMHMM (v2.0) (13), and SOSUI (14) were utilized. tRNAscan-SE (v2.0) (15) and ARAGORN (v1.2.41) (16) were utilized to identify putative tRNAs and transfer-messenger RNAs. The data for VanLee are archived in Phamerator (17). 

**FIG 1:**
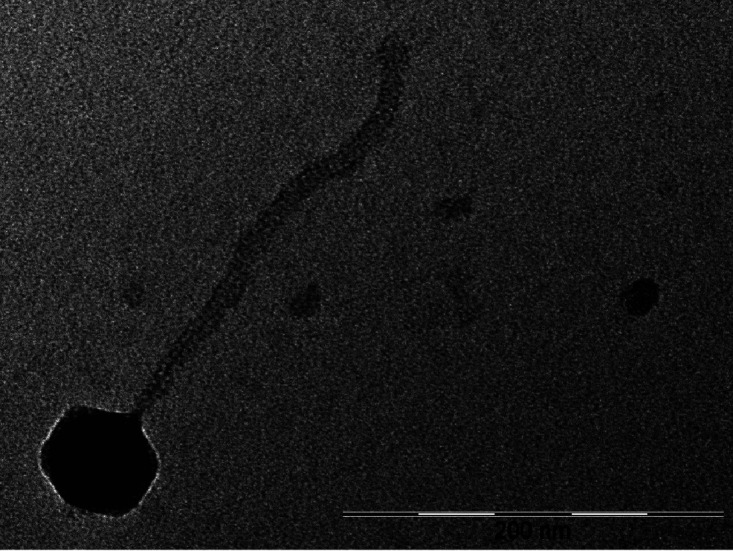
Transmission electron micrograph of *Gordonia* phage VanLee (https://phagesdb.org/phages/VanLee). Phage lysates were negatively stained with 1% uranyl acetate. Scale bar = 200 nm.

Negative-staining transmission electron microscopy shows that VanLee is a siphovirus and a putative member of the family *Siphoviridae*, with an icosahedral capsid of ∼60 nm and a 240-nm tail (Fig. 1). VanLee has an 84,560-bp genome, has a GC content of 67.8%, and contains 164 predicted protein-coding genes and one tRNA (Arg [TCT]). Eighteen of the protein-coding genes are predicted to encode structural proteins, with an additional 34 genes predicted to encode enzymes or DNA-binding proteins. VanLee has <67% average nucleotide identity (ANI) to other phages in the Actinobacteriophage Database, as determined by OrthoANI (18), and is classified as a singleton. Seventy-three of the predicted genes in VanLee do not encode proteins that have homologues among known actinobacteriophages or other organisms, as evaluated using NCBI BLAST (11) or HHpred (v3.2) (10). VanLee infection results in turbid plaques, suggesting that it is a temperate phage. Consistent with this observation, VanLee has an immunity cassette containing a tyrosine integrase (gp32), immunity repressor (gp34), control of repressors operator, Cro (gp35), and excise (gp40). VanLee also contains both HicB-like and HicA-like toxin/antitoxin genes (19). Finally, serial dilutions of VanLee lysates show identical infection efficiencies when Gordonia rubripertincta NRRL B-16540 and Gordonia terrae 36212 are used as hosts.

### Data availability.

Data for VanLee are archived in the Actinobacteriophage Database (3) (https://phagesdb.org/phages/VanLee). This whole-genome shotgun project has been deposited in DDB/ENA/GenBank under the accession no. MZ028627 and SRX11195424. The version described in this paper is the first version.
